# *Toxoplasma gondii* inhibits cytochrome *c*-induced caspase activation in its host cell by interference with holo-apoptosome assembly

**DOI:** 10.15698/mic2015.05.201

**Published:** 2015-05-04

**Authors:** Kristin Graumann, Frieder Schaumburg, Thomas F. Reubold, Diana Hippe, Susanne Eschenburg, Carsten G. K. Lüder

**Affiliations:** 1 Institute for Medical Microbiology, Georg-August-University, Göttingen, Germany.; 2 Institute for Biophysical Chemistry, Hannover Medical School, Hannover, Germany.; 3 Present address: In den Brühlwiesen 12, 61352 Bad Homburg, Germany.; 4 Present address: Institute for Medical Microbiology, University Hospital Münster, Domagkstraße 10, 48149 Münster, Germany.

**Keywords:** Toxoplasma gondii, apoptosis, pathogen-host interaction, caspase activation, intrinsic pathway, apoptosome, Apaf-1

## Abstract

Inhibition of programmed cell death pathways of mammalian cells often facilitates the sustained survival of intracellular microorganisms. The apicomplexan parasite *Toxoplasma gondii* is a master regulator of host cell apoptotic pathways. Here, we have characterized a novel anti-apoptotic activity of *T. gondii*. Using a cell-free cytosolic extract model, we show that *T. gondii* interferes with the activities of caspase 9 and caspase 3/7 which have been induced by exogenous cytochrome *c* and dATP. Proteolytic cleavage of caspases 9 and 3 is also diminished suggesting inhibition of holo-apoptosome function. Parasite infection of Jurkat T cells and subsequent triggering of apoptosome formation by exogenous cytochrome *c* *in vitro* and *in vivo* indicated that *T. gondii* also interferes with caspase activation in infected cells. Importantly, parasite inhibition of cytochrome* c*-induced caspase activation considerably contributes to the overall anti-apoptotic activity of *T. gondii* as observed in staurosporine-treated cells. Co-immunoprecipitation showed that *T. gondii* abolishes binding of caspase 9 to Apaf-1 whereas the interaction of cytochrome *c *with Apaf-1 remains unchanged. Finally, *T. gondii* lysate mimics the effect of viable parasites and prevents holo-apoptosome functionality in a reconstituted *in vitro* system comprising recombinant Apaf-1 and caspase 9. Beside inhibition of cytochrome *c* release from host cell mitochondria, *T. gondii* thus also targets the holo-apoptosome assembly as a second mean to efficiently inhibit the caspase-dependent intrinsic cell death pathway.

## INTRODUCTION

*Toxoplasma gondii* is an intracellular protozoan parasite which is ubiquitous throughout the world and which infects a broad range of mammals and birds including up to one third of the human population. Although infection of immunocompetent individuals is usually asymptomatic or benign, it can lead to significant illnesses including lymphadenopathy or ocular disease in some patients. In addition, *T. gondii* is a major opportunistic and life-threatening pathogen of immunocompromized patients and of fetuses after trans-placental transmission 1. Following ingestion of the parasite via contaminated food or water, or after uptake from the environment, fast replicating tachyzoites disseminate within the host. They partially transform to dormant bradyzoites which are able to persist within tissue cysts for the host’s life time. Long-term persistence is one of the hallmarks of *T. gondii* infection and is critical for parasite transmission and pathogenesis of reactivated toxoplasmosis.

*T. gondii* invades its host cell by active penetration through a moving junction at the host cell surface 2. This enables the parasite to infect essentially any cell type of warm-blooded vertebrates. It leads to formation of a parasitophorous vacuole (PV) which is extensively modified by the parasite. During and after invasion, *T. gondii* secretes a variety of virulence factors mainly from two types of excretory-secretory organelles, namely the rhoptries and the dense granules 3,4. These proteins are in part directly injected into the host cell cytosol during host cell penetration, or they are translocated to and inserted into the PV membrane where some of them have access to host cell signaling components 3,5,6. Rhoptry and dense granule proteins have been recognized as microbial master regulators of the host cell physiology which are crucial for intracellular survival of *T. gondii*.

One of the innate resistance mechanisms of higher eukaryotes against intracellular pathogens such as *T. gondii* is the triggering of programmed cell death (PCD) 7,8,9. This includes the execution of the intrinsic ‘suicide’ program induced by intracellular infection in order to restrict further development of the invader 10. In addition, inflammatory responses during acute *T. gondii* infection lead to activation-induced PCD 11,12,13. PCD can be induced after activation of cell surface receptors including Fas/CD95, after perforin-mediated uptake of granzyme B, or after encountering cellular stressors, e.g. radiation, growth factor deprivation or infection (reviewed in 14). The cell-intrinsic PCD pathway converges at the level of pro- and anti-apoptotic proteins of the Bcl-2 family which transduce death-promoting signals into the permeabilization of the outer mitochondrial membrane (MOMP) 15. It is also fuelled after triggering Fas/CD95 of type II cells 16 indicating a critical role of Bcl-2 proteins and MOMP during extrinsic death receptor-mediated PCD as well 17. MOMP leads to the release of apoptogenic proteins including cytochrome *c* from mitochondria into the cytosol where it binds to the regulatory WD40 repeat domain at the COOH-terminus of the apoptotic protease activating factor 1 (Apaf-1) 18,19,20,21. In the presence of ATP or dATP, cytochrome *c*-binding induces a conformational change in Apaf-1 which then allows Apaf-1 to form a wheel-like heptameric complex called the apoptosome 21. Binding of caspase 9 to Apaf-1 via CARD (caspase recruitment domain)-CARD interaction leads to formation of the holo-apoptosome. Caspase 9 is then activated and subsequently cleaves downstream effector caspases 3, 6 and 7 22. Activation of effector caspases is believed to represent a ‘point-of-no-return’ that leads to execution of apoptosis, i.e. a caspase-dependent form of PCD 23.

Various intracellular pathogens including *T. gondii* have evolved mechanisms to inhibit PCD of their host cells (reviewed in 24,25). Interference with host cell PCD signaling pathways at least prolongs the viability of the host cell by inhibiting cell-intrinsic or extrinsic death-receptor mediated PCD and thereby facilitates pathogen survival. Genetically modified malaria parasites and mycobacteria that are unable to inhibit caspase-dependent PCD within their host cells are indeed rapidly cleared after infection 26,27. Infection with *T. gondii* renders mammalian cells largely resistant to the caspase-dependent intrinsic PCD triggered by irradiation, growth factor withdrawal and treatment with different cytotoxic agents 28,29,30,31,32,33. It is believed that anti-apoptotic activities of *T. gondii* also counteract the innate PCD program with which infected host cells would normally respond to infection 10,28,33,34. Importantly, during *Toxoplasma* encephalitis in mice, parasite-infected host cells are also protected from undergoing inflammation-associated PCD 35,36. Release of cytochrome *c* from mitochondria to the host cell cytosol is profoundly decreased in parasite-positive cells 30,32 and this is at least in part due to reduced activation of the pro-apoptotic Bcl-2 effector protein Bax 37. Activation of NF-κB 31,38 and protein kinase B/Akt 33 may function as upstream signaling pathways contributing to the block of caspase-dependent intrinsic cell death in infected cells. By using a cell-free *in vitro* system of caspase activation, we have recently shown that *T. gondii* or excretory-secretory proteins released by the parasite are able to inhibit cytochrome *c*-triggered activation of caspase 3/7 39. Since this inhibition occurs in the absence of intact host cell mitochondria, it clearly differs from the well-known inhibition of mitochondrial cytochrome* c* release exerted by *T. gondii*. In this study, we have pinpointed the holo-apoptosome formation as the step of cytochrome *c*-mediated caspase activation that is abrogated by *T. gondii*. Importantly, the data suggest that this mechanism contributes to a similar extent to the overall inhibition of the caspase-dependent intrinsic PCD pathway as the inhibition of MOMP and cytochrome *c* release from mitochondria of infected host cells.

## RESULTS

### Impact of *T. gondii* on cytochrome *c*-induced caspase activation in cytosolic Jurkat extracts

**Figure 1:**
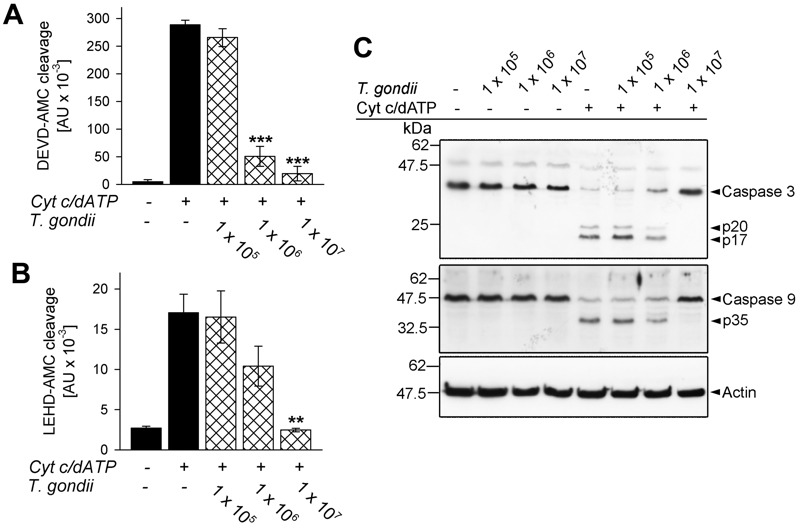
FIGURE 1: *T. gondii* inhibits cytochrome *c*-induced activation of the caspase 9-caspase3/7 cascade in cell-free cytosolic extracts. **(A, B) ** Cell-free cytosolic extracts of Jurkat cells were incubated with increasing amounts of parasites as indicated (cross-hatched bars; no. of parasites per 0.1 ml) or were left untreated (black bars). After 1 hour, caspase activation was triggered or not by cytochrome *c* and dATP. Cleavage of the caspase 3/7 substrate DEVD-AMC **(A)** or of the caspase 9 substrate LEHD-AMC **(B) ** was measured fluorimetrically. Data represent the increase in substrate cleavage over time; they represent means ± S.E.M. from at least 3 independent experiments. Significant differences were identified by Student’s *t*-test (** P < 0.01; *** P < 0.001). **(C) ** Cell-free cytosolic extracts incubated or not with *T. gondii* as indicated and incubated or not with cytochrome *c*/dATP to trigger caspase activation were resolved by SDS-PAGE. After protein transfer to nitrocellulose, membranes were probed with antibodies recognizing caspase 3, caspase 9 and actin. Immune complexes were visualized using peroxidase-conjugated secondary antibodies and enhanced chemiluminescence detection. The experiment was repeated once with similar results.

Cytosolic extracts of Jurkat cells have been widely used to dissect activation of the caspase cascade by cytochrome *c* independently of the release of PCD-regulating proteins following MOMP 39. Incubation of such extracts with *T. gondii* prior to the addition of cytochrome *c* and dATP dose-dependently inhibited caspase 3/7 activity as determined by decreased cleavage of the fluorogenic peptide-based substrate Ac-Asp-Glu-Val-Asp-7-amino-4-methylcoumarin (DEVD-AMC) (Fig. 1A). This confirms a previous report that *T. gondii* is able to directly interfere with cytochrome *c*-induced caspase 3/7 activation independently of the inhibition of MOMP 39. In order to get further insights into this novel putative anti-apoptotic activity of *T. gondii* we determined the activity of the initiator caspase 9 by measuring cleavage of the fluorogenic peptidyl substrate Ac-Leu-Glu-His-Asp-AMC (LEHD-AMC). Cytochrome* c* in the presence of dATP induced LEHDase activity in cytosolic Jurkat extracts whereas cleavage was dose-dependently inhibited by *T. gondii* (Fig. 1B). Indeed, 1 × 10^8^ parasites per ml completely abrogated cytochrome *c*/dATP-induced caspase 9 activity (P = 0.003; Student’s *t*-test) similar to what we observed for caspase 3/7 activity.

Caspases are activated upon cleavage of the inactive zymogens into distinct subunits which subsequently assemble into the mature heteromultimers. In cytosolic Jurkat extracts incubated with *T. gondii*, however, proteolytic processing of inactive procaspase 3 into p20 and p17 and of procaspase 9 into p35 induced by cytochrome *c*/dATP was dose-dependently inhibited by the parasite (Fig. 1C) and was completely abolished by 1 × 10^8^ parasites per ml of cytosolic host cell extract. Thus, *T. gondii* is able to inhibit activation of the caspase 9-caspase 3 cascade independently of its effect on MOMP 30,37.

### Inhibition of cytochrome *c*/dATP-induced caspase activation in *T. gondii*-infected cells

**Figure 2:**
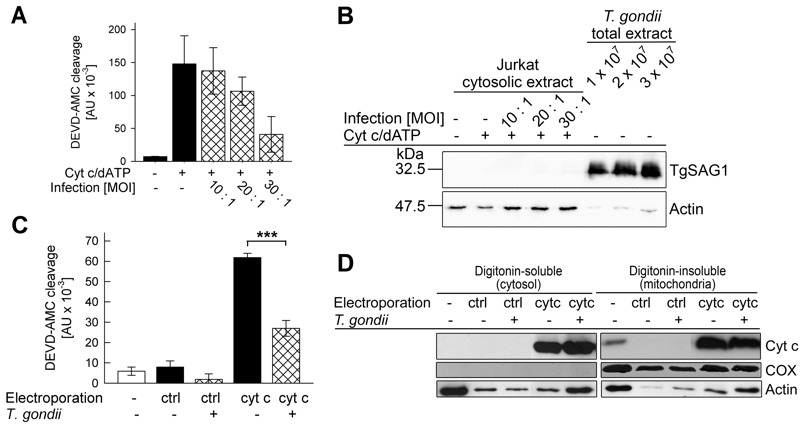
FIGURE 2: Infection of host cells with *T. gondii* diminishes *in vitro* and *in vivo* activity of caspase 3/7 triggered by exogenous cytochrome *c*. **(A, B) ** Jurkat T cells were infected with increasing amounts of *T. gondii* (multiplicity of infection (MOI) 10:1 to 30:1; cross-hatched bars or as indicated) or were left non-infected (black bars or as indicated). **(A) ** After 24 hours of infection, cytosolic extracts were isolated and the caspase cascade was activated using cytochrome *c*/dATP. Caspase 3/7 activity was determined by fluorimetric measurement of DEVD-AMC cleavage over time. Results represent means ± S.E.M. (n=3). **(B) ** Alternatively, the cytosolic extracts from Jurkat cells and total extract from the same numbers of parasites as having been used for infection were separated by SDS-PAGE and analyzed by immunoblotting using antibodies which recognize *T. gondii* SAG1 or host cell actin. Bound antibodies were visualized using peroxidase-conjugated secondary antibodies and enhanced chemiluminescence detection. Results are representative for two independent experiments. **(C) ** Jurkat cells infected with *T. gondii* for 1 hour (MOI 30:1; cross-hatched bars) and non-infected control cells (open bar and black bars) were electroporated in the presence of cytochrome *c* (cyt*c*) or bovine serum albumin (ctrl) or were left non-treated. After incubation of the cells and subsequent cell lysis, caspase activity was determined by fluorimetric measurement of the cleavage of the caspase 3/7 substrate DEVD-AMC. Data represent the increase of cleavage over time; bars indicate means ± S.E.M. (n=6). Significant differences were identified by Student’s *t*-test (*** P < 0.001). **(D) ** After infection and/or electroporation of cells as described above (C), cells were partitioned into a digitonin-soluble fraction comprising the host cell cytosol and a digitonin-insoluble fraction including mitochondrial proteins. Proteins were resolved by SDS-PAGE and after transfer to nitrocellulose, were immunolabelled using antibodies recognizing cytochrome *c* (cyt *c*), cytochrome *c*-oxidase subunit IV (COX) or actin. Immune complexes were visualized using peroxidase-conjugated secondary antibodies and enhanced chemiluminescence detection. Similar results were obtained in two independent experiments.

In infected cells, *T. gondii* resides inside a membrane-bound compartment, i.e. the parasitophorous vacuole, which restricts the direct access of parasite components to signaling pathways of the host. It was therefore critical to assess whether *T. gondii* also directly interferes with cytochrome *c*-induced caspase activation when being confined to its natural intracellular habitat. In order to distinguish between the parasite-mediated inhibition of cytochrome *c* release from mitochondria as described previously 30,37 and the mechanism described herein in infected cells, we measured the caspase 3/7 activity which had been induced in cytosolic extracts from non-infected and *T. gondii*-infected Jurkat cells after addition of cytochrome *c *and dATP. The results showed that infection with *T. gondii* for 24 hours dose-dependently reduced such DEVD-AMC cleavage activity (Fig. 2A) suggesting parasite interference with caspase activation independently of MOMP in infected cells.

In order to exclude a contamination of the cytosolic Jurkat extracts with cell-associated parasite proteins during the extraction procedure, cytosolic extracts from infected and non-infected Jurkat cells were analyzed by SDS-PAGE and immunoblotting. The major surface antigen (SAG)-1 of *T. gondii* was hardly detected in the lysate of Jurkat cells which had been infected with *T. gondii* even at the highest MOI (Fig. 2B). In contrast, TgSAG1 was strongly present in total lysates from the same numbers of parasites as used for infection. Control staining using an anti-actin antibody confirmed equal loading of lysates from infected and non-infected Jurkat cells onto the gel (Fig. 2B). Whether the faint actin bands, as observed in the total parasite extracts, are due to a cross-reaction of the antibody with parasite actin or a contamination of the isolated parasites with host cells is unknown. Together, these data strongly suggest that *T. gondii* can indeed inhibit cytochrome *c*-triggered caspase activation in infected cells.

In order to corroborate these data, we next electroporated *T. gondii*-infected and non-infected Jurkat cells in the presence of exogenous cytochrome *c* and subsequently compared the caspase 3/7 activity in extracts derived from these cells. Immunoblotting confirmed that electroporation in the presence of cytochrome *c* but not in the presence of a control protein (bovine serum albumin, BSA) strongly increased the amount of cytochrome *c* in the digitonin-soluble, i.e. the cytosolic fraction of Jurkat cells irrespective of whether being infected with *T. gondii* or not (Fig. 2D). The amount of cytochrome *c* also strongly increased in the digitonin-insoluble mitochondria-containing fraction after electroporation and clearly exceeded that of untreated control cells (Fig. 2D). Control staining with a cytochrome c-oxidase-specific antibody confirmed complete partitioning of mitochondria into the digitonin-insoluble fraction. Furthermore, staining of actin suggested a general loss of intact cells after electroporation as expected (Fig. 2D).

Most importantly, despite similar amounts of cytosolic cytochrome *c* in *T. gondii*-infected and non-infected cells after electroporation, the DEVD-AMC cleavage activity was significantly lower in infected cells as compared to non-infected cells (Fig. 2C; P = 0.000013; Student’s *t*-test). Electroporation in the presence of the control protein BSA did not increase the DEVDase activity in both non-infected and infected cells (Fig. 2C) thus confirming the specificity of the cytochrome *c*-mediated activity and its inhibition by *T. gondii*.

Together, these data indicate that *T. gondii* interferes with activation of the caspase 9-caspase 3 cascade both in infected cells and cell-free systems. Furthermore, this inhibition differs from the inhibition of mitochondrial cytochrome *c*-release as observed in *T. gondii*-infected cells.

**Figure 3:**
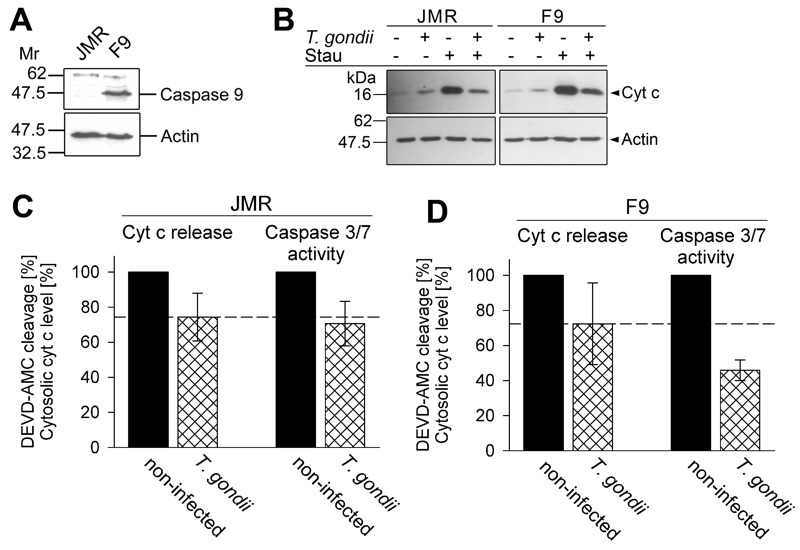
FIGURE 3: Total inhibition of the intrinsic apoptotic pathway by *T. gondii* considerably relies on a mechanism that operates down-stream of mitochondrial cytochrome *c* release and which relies on caspase 9. **(A) ** Total cell lysates were prepared from caspase 9-deficient Jurkat cells (clone JMR) and a reconstituted mutant thereof (F9) and were separated by SDS-PAGE. After protein transfer, nitrocellulose membranes were probed with antibodies recognizing caspase 9 and actin. Immune complexes were visualized using peroxidase-conjugated secondary antibodies and enhanced chemiluminescence. **(B) ** JMR and F9 cells were infected with *T. gondii* for 24 hours (MOI 20:1) or were left non-infected and were then treated or not with staurosporine to trigger the cell-intrinsic PCD pathway. After 90 minutes, cytosolic proteins were isolated by digitonin lysis (digitonin-soluble extract). Proteins were resolved by SDS-PAGE and after transfer to nitrocellulose, were probed with antibodies recognizing cytochrome *c* (cyt *c*) or actin. Bound antibodies were visualized using peroxidase-conjugated secondary antibodies and enhanced chemiluminescence. Band intensities of cytosolic cytochrome *c* after treatment of cells with staurosporine were determined by densitometric analysis and were normalized to actin band intensities. **(C, D) ** Cells were infected with *T. gondii* and/or treated with staurosporine as described above (B). After cell lysis, cleavage of the caspase 3/7 substrate DEVD-AMC was measured fluorimetrically. Data represent the increase of cleaved substrate over time. For comparison, the levels of cytosolic cytochrome *c *as detemined by densitometric analysis (B) are displayed. Results are expressed as mean percentages ± S.E.M. of at least three independent experiments; the caspase 3/7 activities and the levels of cytosolic cytochrome *c* in non-infected cells have been set to 100%.

In order to assess the relative contribution of the parasites’ inhibition of cytochrome *c*-induced caspase activation as compared to the inhibition of MOMP to the overall inhibition of caspase-dependent intrinsic PCD exerted by *T. gondii*, we employed the caspase 9-deficient Jurkat line JMR and a genetically reconstituted mutant thereof (F9) 40. Immunoblotting confirmed expression of caspase 9 in the complemented F9 but not the parental JMR line (Fig. 3A). Treatment with the pro-apoptotic kinase inhibitor staurosporine led to a similar release of cytochrome *c* into the cytosol in both cell lines as determined by immunoblotting of the digitonin-soluble cytosolic fractions (Fig. 3B). Furthermore, infection with *T. gondii* inhibited cytochrome *c* release by ~25% in both cell lines as determined by densitometric analyses (Fig. 3C, D). Most importantly, caspase 3/7 activity was further reduced in *T. gondii*-infected F9 cells to 46% of non-infected control cells (Fig. 3D). As expected, the caspase 3/7 activity in the caspase 9-deficient JMR cells was much lower (non-infected: 4,650 ± 1,092; infected: 3,085 ± 611 [mean AU ± SEM]) as compared to that in caspase 9-proficient F9 cells (non-infected: 163,391 ± 10,361; infected: 74.802 ± 10,093). Cell-intrinsic caspase-dependent PCD can occur in the absence of caspase 9 or Apaf-1 and has been suggested to depend on caspase 7 41 which may explain the residual DEVDase activity as observed in JMR cells. Remarkably, the overall low caspase 3/7 activity in caspase 9-deficient cells was not further inhibited by *T. gondii* infection when compared to the parasite-mediated inhibition of cytochrome *c* release (Fig. 3C). This strongly suggests that the additional inhibition of the caspase 3/7 activity in F9 cells, as compared to the inhibition of mitochondrial cytochrome *c* release, is due to parasite interference with the caspase 9-caspase 3 pathway. Thus, ~50% of the total parasite inhibition of caspase 3/7 activity after triggering the intrinsic apoptotic pathway may be achieved by interference of *T. gondii* with the activation of caspase 9.

### *T. gondii* inhibits holo-apoptosome formation but not cytochrome *c*-Apaf-1 binding

**Figure 4:**
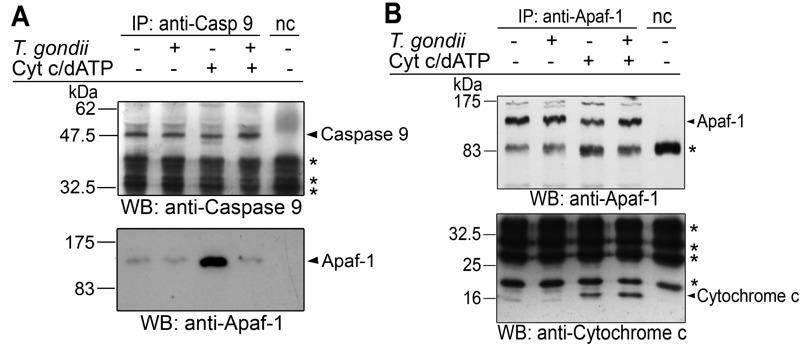
FIGURE 4: Interference of *T. gondii* with holo-apoptosome assembly as revealed by co-immunoprecipitation analyses. **(A, B) ** Cell-free cytosolic extracts from Jurkat cells were incubated or not with *T. gondii* (10^8^/ml). After 1 hour, apoptosome formation was triggered by addition of cytochrome *c* and dATP as indicated. Caspase 9 **(A) ** or Apaf-1 **(B) ** was immunoprecipitated using specific antibodies and protein A-sepharose in the presence of a caspase 3-inhibitor. A negative control precipitation without cell lysate was run in parallel (nc). Precipitates were resolved by SDS-PAGE and were analyzed by immunoblotting using specific antibodies as indicated. Bound antibodies were visualized by enhanced chemiluminescence after incubation with appropriate peroxidase-conjugated secondary antibodies. Unspecific binding of secondary antibodies is indicated by asterisks. The experiment was repeated twice with similar results.

In order to further elucidate the mechanism of the parasite-mediated inhibition of caspase 9 activation, we performed co-immunoprecipitation analyses of components of the holo-apoptosome. To this end, cytosolic Jurkat extracts were incubated with or without *T. gondii* and formation of the apoptosome was induced by cytochrome *c* and dATP. Caspase 9 was immunoprecipitated from all cell lysates to a similar extent irrespective of whether having been incubated with *T. gondii* and/or treated with cytochrome *c*/dATP or not (Fig. 4A). Addition of cytochrome *c* and dATP to Jurkat lysate triggered the formation of caspase 9-Apaf-1 complexes, i.e. holo-apoptosome formation. Remarkably, *T. gondii* at a concentration of 1 × 10^8^ parasites per ml lysate completely abolished co-immunoprecipitation of Apaf-1, indicating that *T. gondii* inhibits the cytochrome *c*-triggered complex formation (Fig. 4A).

After immunoprecipitation of Apaf-1, similar amounts of 130 kDa full length Apaf-1 were pulled down from all experimental samples but not from a negative control without cytosolic Jurkat extract (Fig. 4B). After triggering apoptosome formation, equal amounts of cytochrome *c *were co-precipitated with Apaf-1 irrespective of whether Jurkat lysates had been preincubated with *T. gondii* or not. This indicates that *T. gondii* does not interfere with one of the initial steps of apoptosome formation, i.e., binding of cytochrome *c*.

Together, these data suggest that *T. gondii* prevents the interaction of caspase 9 with Apaf-1, i.e. a critical step in the formation of the holo-apoptosome.

### *T. gondii* protein extract inhibits caspase 9 activation in an *in vitro* reconstituted apoptosome formation system

In order to further pinpoint the interference of *T. gondii* with the apoptosome and to exclude any bystander effects of components of the Jurkat cytosol, an *in vitro* apoptosome assembly system was employed. The reconstituted system consisted of purified Sf21 insect cell-derived recombinant Apaf-1, purified *Escherichia coli*-derived recombinant full-length caspase 9 and horse heart cytochrome *c* as described previously 21. In order to also corroborate the hypothesis that a parasite effector directly interacts with one of these apoptosome components without the necessity of viable parasites 39, we used a PBS-soluble *Toxoplasma* lysate in these experiments.

**Figure 5:**
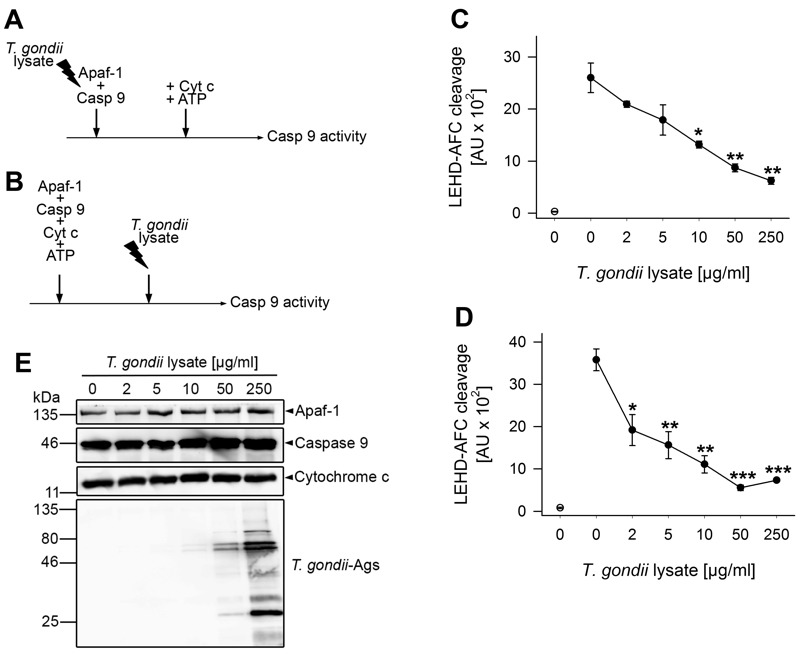
FIGURE 5: *T. gondii* protein extract dose-dependently diminishes caspase 9 activity triggered by cytochrome *c* and ATP in a reconstituted *in vitro* system. **(A, C) ** Recombinant human Apaf-1 and caspase 9 were incubated with increasing amounts of *T. gondii* protein lysate or were left untreated. After 1 hour, apoptosome formation was triggered by adding cytochrome *c* and ATP, and 15 minutes later, caspase 9 activity was determined by fluorimetric measurement of LEHD-AFC cleavage. **(B, D) ** Recombinant Apaf-1 and caspase 9 were induced to form apoptosomes using cytochrome *c* and ATP. After 1 hour, preassembled apoptosomes were incubated with increasing amounts of *T. gondii* protein extract for 45 minutes. Fifteen minutes later, caspase 9 activity was fluorimetrically measured as above. **(C, D) ** Data represent the increase in substrate cleavage over time from samples as described in (A, B); results are expressed as means ± S.E.M. (n = 3). Background activity in samples without cytochrome *c*/ATP is indicated by an open circle. Significant differences between activated samples without *T. gondii* proteins and those incubated with *T. gondii* proteins have been identified by Student’s *t*-test (* P < 0.05; ** P < 0.01; *** P < 0.001). **(E) ** Recombinant and/or purified components were incubated with increasing amounts of *T. gondii* lysate according to the protocol depicted in (A) but without adding ATP. After SDS-PAGE and Western blotting, proteins were analyzed by immunostaining using specific antibodies as indicated and peroxidase-conjugated secondary antibodies. Immune complexes were visualized by enhanced chemiluminescence. Results are representative for two experiments.

Incubation of Jurkat cytosolic extract with 2 to 10 µg/ml of parasite proteins confirmed previous results 39 that *Toxoplasma* lysate does suffice to inhibit cytochrome *c*/dATP-triggered caspase 3/7 activity (Fig. S1A). Furthermore, 2 to 10 µg/ml parasite lysate also clearly inhibited caspase 9 activity although this did not reach statistical significance due to a higher background activity and some variability between experiments (Fig. S1B). Importantly, incubation of purified Apaf-1 and caspase 9 together with *T. gondii* extract dose-dependently prevented subsequent activation of caspase 9 triggered by cytochrome *c* and ATP (Fig. 5A, C). LEHD-AFC (AFC: 7-amino-4-trifluoromethylcoumarin) cleavage was significantly inhibited after adding 10 µg/ml *T. gondii* proteins (P = 0.012; Student’s *t*-test) and higher concentrations even further decreased substrate cleavage (P = 0.004 (50 µg) and P = 0.002 (250 µg); Student’s *t*-test). In the absence of cytochrome *c*/ATP, only background caspase 9 activity was observed (Fig. 5C).

In order to exclude the possibility that inhibition of caspase 9 activation resulted from an unspecific proteolytic activity of the parasite lysate, immunoblot analyses were performed. To this end, recombinant Apaf-1, caspase 9 and purified cytochrome *c* were incubated with increasing amounts of *T. gondii* lysate according to the scheme shown in Fig. 5A but in the absence of ATP to avoid auto-proteolysis. The results show that even in the presence of

250 µg/ml parasite lysate, full-length Apaf-1, caspase 9 and cytochrome *c *remained intact during the time of observation (Fig. 5E).

We then addressed the question whether *T. gondii* effector protein(s) also inhibit caspase 9 activity after pre-assembly of apoptosomes (Fig. 5B). Indeed, even when added 1 hour after triggering apoptosome formation, 2 µg/ml parasite lysate significantly decreased LEHD-AFC cleavage (P = 0.021) and higher concentrations of lysate further inhibited caspase activity (Fig. 5D; P = 0.0017 (10 µg) and P = 0.00034 (50 µg)). No LEHD-AFC cleavage occurred in the absence of cytochrome *c*/ATP.

Together, these data indicate that a parasite effector can directly prevent cytochrome *c*-triggered caspase 9 activation both during and after onset of apoptosome assembly.

## DISCUSSION

*T. gondii*-infected cells are surprisingly resistant against induction of PCD *in vitro* and *in vivo* 28,29,30,31,32,33,35,36,37,42. Here, we identify the Apaf-1 apoptosome as a hitherto unrecognized target of *T. gondii* to inhibit caspase-dependent intrinsic host cell death. Using an *in vitro* reconstitution system we exclude any indirect effects of host cell cytosolic components apart from Apaf-1, caspase 9 and cytochrome *c* being involved in the inhibition of cytochrome *c*-triggered caspase 9 activation. Importantly, our data indicate that the novel anti-apoptotic activity significantly contributes to the ability of *T. gondii* to inhibit the caspase-dependent intrinsic apoptotic pathway in infected cells. To the best of our knowledge, this represents the first example of an infectious agent that diminishes host cell apoptosis by interference with cytochrome *c*-induced apoptosome formation.

**Figure 6:**
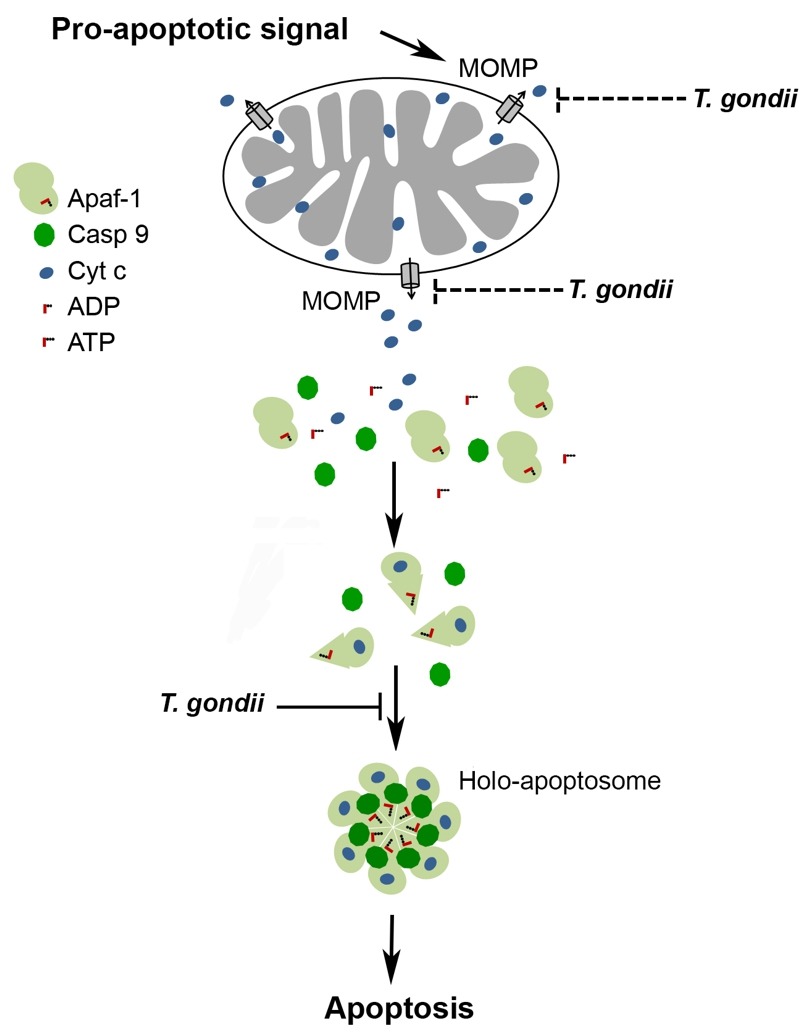
FIGURE 6: Model of the *T. gondii*-mediated inhibition of the caspase-dependent intrinsic PCD pathway. A cell-intrinsic pro-apoptotic signal leads via activation of pro-apoptotic Bcl-2 family members to MOMP and release of apoptogenic molecules including cytochrome *c* from mitochondria. Binding of cytochrome *c* to Apaf-1 and nucleotide exchange induce conformational changes that allow oligomerization of Apaf-1, caspase 9 recruitment and caspase activation. *T. gondii* inhibits the mitochondrial apoptotic pathway at least twofold: (i) by inhibition of MOMP 17,30,32,37 and (ii) by inhibiting cytochrome *c*-triggered holo-apoptosome assembly (described herein). See main text for further details.

After release of cytochrome *c* from mitochondria through MOMP, formation of the apoptosome is pivotal for activating the initiator caspase 9 which subsequently activates effector caspases 3, 6 and 7 43,44. Here we provide clear evidence that *T. gondii* inhibits the binding of caspase 9 to Apaf-1 thereby abrogating caspase 9 activity and subsequent caspase 3/7 activation (Fig. 6). Thus, recruitment of caspase 9 to the N-terminal CARD of Apaf-1 could be inhibited by the parasite. Alternatively, the heptamerization of Apaf-1-cytochrome *c*-dATP complexes can also be hindered by *T. gondii* since formation of the Apaf-1 heteroheptamer may significantly increase caspase 9 binding to Apaf-1 18,19,22. This is reminiscent to previous findings that mammalian heat shock protein (HSP)-70 inhibits apoptosis by preventing recruitment of caspase 9 to Apaf-1 45 and/or formation of Apaf-1 oligomers 46.

Our co-immunoprecipitation assays revealed a basal level of Apaf-1-caspase 9 interaction in the absence of cytochrome *c*/dATP which was strongly increased after addition of both cofactors (see Fig. 4A). This constitutive interaction may be due to the formation of Apaf-1-caspase 9 heterodimers as also indicated by others 47,48,49. Remarkably, formation of these complexes was not inhibited in the presence of the parasite whereas cytochrome *c*/dATP-induced ones were completely abolished by *T. gondii*. This argues for a distinct step of the holo-apoptosome formation rather than mere binding of caspase 9 to monomeric Apaf-1 being inhibited by the parasite. One prerequisite for apoptosome assembly, i.e. binding of cytochrome *c* to Apaf-1, is not abrogated by *T. gondii* (see Fig. 4B). Cytochrome *c* binds to a groove between two regulatory β-propellers of Apaf-1 49. Furthermore, even a ten-fold excess of dATP does not abrogate the ability of *T. gondii* to inhibit cytochrome *c*-triggered caspase activation *in vitro* (Schaumburg and Lüder, unpublished data). It is interesting to note that using an *in vitro *apoptosome reconstitution system, we demonstrate that parasite lysate inhibits caspase 9 activation even when added to the pre-assembled holo-apoptosome. We therefore propose that *T. gondii* can interfere with the function of both monomeric Apaf-1 as well as pre-assembled apoptosomes thereby preventing recruitment of caspase 9 via their CARD and caspase activation (Fig. 6). Further analyses are required to exactly pinpoint the molecular mechanisms of the parasite-Apaf-1 interaction.

Several previous reports have provided unequivocal evidence that infection with *T. gondii* inhibits the release of cytochrome *c* from mitochondria to the host cell cytosol which is one of the pivotal events that regulate the caspase 9/caspase 3/6/7 pathway 17,30,32,37. Using a mitochondria-free cytosolic caspase activation system, we describe herein a different mechanism of *T. gondii* thus confirming previous findings 39. Importantly, using a reconstituted *in vitro* system comprising recombinant Apaf-1 and caspase 9 as well as purified cytochrome *c* 21 and dATP, we can exclude any other host cell cytosolic components being responsible for this parasite-host interaction.

Even more important is the evidence that the mechanism described herein operates in the parasite-infected cell. Firstly, parasite infection of host cells prior to the isolation of cytosolic extracts dose-dependently inhibits activation of the caspase cascade triggered by exogenous cytochrome *c* and dATP. Importantly, a contamination with cell-associated *T. gondii* proteins which could have been released during the Jurkat lysis was largely excluded. Instead, a yet unknown parasite effector which is responsible for abrogating holo-apoptosome formation may reach the host cell cytosol in the infected cell and has access to the apoptosome machinery thereby inhibiting caspase activation. We have shown previously that molecules discharged by extracellular *T. gondii* from their excretory-secretory organelles indeed mediate the inhibition of cytochrome *c*/dATP-triggered caspase activation in cell-free extracts 39. It is thus likely that a parasite effector released by *T. gondii* from the rhoptries or the dense granules during or after host cell invasion 3,4,5,6,50,51 interferes with apoptosome formation.

Secondly, electroporation of infected and control cells in the presence of cytochrome *c* also indicates that intracellular *T. gondii* significantly inhibits caspase activation that has been triggered by exogenous cytochrome *c* (see Fig. 2C). Since the caspase cascade was activated independently of the release of cytochrome *c* from host cell mitochondria, this provides direct evidence for the parasite’s ability to target the cell-intrinsic PCD pathway downstream of cytochrome *c* release from mitochondria. *T. gondii* is not able to directly inhibit or even reverse caspase 3/7 activity 39 and apoptosome formation is unlikely to occur after lysis of the intact cells due to the NP-40 lysis buffer used for cell extraction. Therefore, we again consider an inhibiting effect of parasite components on caspase 3/7 activity after host cell lysis unlikely. The data instead also argue for a parasite interference with cytochrome *c*-triggered caspase activation in infected cells.

Finally, comparison of caspase 9-deficient JMR cells and caspase 9-proficient F9 cells revealed that caspase activation is more efficiently inhibited by *T. gondii* in the F9 cells than in the JMR cells whereas cytochrome *c*-release from mitochondria was similarly inhibited in both cells. This suggests that in F9 cells, *T. gondii* employs an additional anti-apoptotic mechanism that requires the presence of caspase 9 and that differs from the inhibition of cytochrome *c* release from host cell mitochondria. We propose that the relative differences in the parasite-mediated inhibition of caspase 3/7 activities between JMR and F9 cells are indeed due to an apoptosome-dependent caspase 9 activation in F9 but not in JMR cells. Importantly, this additional layer of anti-apoptotic activity appears to similarly contribute to the overall inhibition of the caspase-dependent intrinsic PCD pathway by *T. gondii* as the cytochrome *c *release from host cell mitochondria.

Together, this establishes the intriguing fact that *T. gondii* has evolved at least two different mechanisms to prevent the intrinsic apoptotic pathway, namely (i) inhibition of cytochrome *c* release from host cell mitochondria, and (ii) interference with holo-apoptosome assembly once cytochrome *c* has been released from the mitochondria (Fig. 6). Evolution of two layers of anti-apoptotic mechanisms by *T. gondii* within the same pathway may provide a safeguard to guarantee efficient abrogation of cell-intrinsic apoptosis thereby ensuring parasite survival.

## MATERIALS AND METHODS

### Cell lines and parasite strain

Jurkat E6.1 human-derived leukemic T cells (European Collection of Animal Cell Cultures, Salisbury, UK) were cultivated in RPMI 1640 supplemented with 10% heat-inactivated fetal calf serum (FCS), 100 U/ml penicillin and 100 µg/ml streptomycin. A caspase 9-deficient Jurkat clone (JMR), and a caspase 9-reconstituted mutant thereof (F9) were kindly provided by Ingo Schmitz (Braunschweig, Germany) and have been described previously 17,40. Tachyzoites of the *T. gondii* type II strain NTE 52 were propagated in L929 murine fibroblasts as host cells in RPMI 1640, 1% FCS and antibiotics as above. Parasites were separated from host cells by differential centrifugation as described 42.

### Preparation of *Toxoplasma* lysate

For large scale production of *T. gondii*, parasites were harvested from human foreskin fibroblasts after host cell lysis and were separated from contaminating host cells by filtration through a 3.0 µm Isopore filter (Merck Millipore, Schwalbach, Germany). After extensive washing, 4 × 10^8^ parasites per ml PBS were lysed by three freeze-thaw cycles and were then sonicated twice on ice for 10 min each at an output level of 15-20% and with a duty cycle of 30% (Branson Sonifier 250, Danbury, CT). Insoluble material was removed by centrifugation at 20,800 × g for 20 min at 4°C, and PBS-soluble proteins were stored at -80°C. The protein concentration was determined by the BCA assay as recommended by the manufacturer (Biorad, München, Germany)

### Parasite infection and triggering of caspase-dependent intrinsic PCD

Wild-type or mutant Jurkat cells were infected with *T. gondii* for 24 hours at infection rates of 5 to 30 parasites per host cell as indicated. The intrinsic PCD pathway in JMR and F9 mutants was induced by treatment with 1 µM staurosporine during the final 90 to 120 min of cultivation. Thereafter, Jurkat cells were washed in PBS and they were then lysed at 2 × 10^7^ cells per ml in 1% NP-40, 150 mM NaCl, 50 mM Tris-HCl, pH 8.0 for 15 min on ice. After centrifugation at 20,800 × g and 4°C for 5 min, caspase activity in the soluble fraction was measured as described below.

### Electroporation of Jurkat cells

The impact of parasite infection on cytochrome *c*-induced caspase activation in infected cells was determined in Jurkat cells which had been electroporated in the presence of cytochrome *c* 53. To this end, 3 × 10^6^ Jurkat cells were infected with *T. gondii* at a MOI of 30:1 for one hour or were left non-infected and were then resuspended in 400 µl FCS-free medium supplemented with 400 µg/ml cytochrome *c* from bovine heart (Sigma-Aldrich, Taufkirchen, Germany) or bovine serum albumin (BSA; Sigma-Aldrich) as a control. Cells were electroporated using a BTX cell manipulator 600 (BTX, San Diego, CA) at 160 V and 2300 µF. Cells were then incubated for 15 min on ice before being incubated in fresh cell culture medium for 60 min at 37°C and 5% CO_2_. Thereafter, cell extracts were prepared by NP-40 lysis (see above) and caspase activity was determined.

### Cell fractionation

The subcellular distribution of cytochrome *c* and control proteins was determined after fractionation of cells into digitonin-soluble and digitonin-insoluble extracts 37. Briefly, 4 × 10^6^ cells per sample were resuspended in 30 µl of PBS and were then mixed with an equal volume of 150 µg/ml digitonin in 500 mM sucrose. After 60 seconds, heavy organelles including mitochondria were pelleted at 14,000 × g for 1 min and supernatants were saved as the cytosol-enriched digitonin-soluble fraction. The digitonin-insoluble fraction was then extracted in 1% Triton X-100, 0.5% sodium deoxycholate, 0.1% SDS, 150 mM NaCl, 50 mM Tris-HCl, pH 8.0, 1 mM PMSF, 1 mM sodium orthovanadate, and 10 µg/ml each of leupeptin, aprotinin and pepstatin. After centrifugation at 20,800 × g and 4°C for 1 min, the supernatant was removed as digitonin-insoluble, mitochondrial proteins-containing extract.

### Caspase activation in cytosolic Jurkat protein lysates

Cytosolic caspase activation extracts of Jurkat T cells were used in order to determine the effect of *T. gondii* on the caspase 9-caspase 3/7 cascade in a cell-free *in vitro* system 39. To this end, Jurkat cells were washed in PBS and were then incubated at 1 × 10^8^ cells per ml in 20 mM HEPES-KOH, pH 7.0, 10 mM KCl, 1 mM EDTA, 1 mM EGTA, 1.5 mM MgCl_2_, 2 mM dithiothreitol (DTT) and protease inhibitor cocktail (EDTA-free; Roche, Mannheim, Germany) (caspase 3 activation). Alternatively, they were incubated at 2 × 10^8^ cells per ml in 50 mM HEPES-KOH, pH 7.5, 50 mM KCl, 0.2% CHAPS, 2 mM MgCl_2_, 5 mM EGTA, 10 µg/ml cytochalasin B, 1 mM DTT and protease inhibitor cocktail as above (caspase 9 activation). After 30 min on ice, cells were lysed by repeated 23G needle passage. Lysis of at least 90% of the cells was controlled by trypan blue staining. The extract was then centrifuged at 10,000 × g and 4°C for 10 min and the cytosol-enriched supernatant stored at -80°C until further use. The protein content of cell lysates was determined by the BCA test.

To determine the impact of *T. gondii* parasites on caspase activation *in vitro*, cytosolic Jurkat extracts were incubated or not with different numbers of parasites or with *T. gondii* lysate for 1 hour at room temperature. Thereafter, the caspase cascade was activated by addition of 10 µg/ml cytochrome *c*, 250 µM dATP and 250 µM DTT or the caspase cascade was left non-activated. After 1 hour at 37°C, parasites were pelleted by centrifugation at 20,800 × g for 5 min and the supernatants were assayed for caspase activities or by immunoprecipitation. In some experiments, the caspase cascade was activated in cytosolic caspase activation extracts which have been isolated from *T. gondii*-infected and non-infected Jurkat cells at 24 hours after infection.

### Caspase activity tests

Caspase activities were measured as described 39,54 in NP-40 lysates from cells triggered to undergo apoptosis or in cell-free cytosolic Jurkat extracts after cytochrome *c*/dATP-induced activation of the caspase cascade. Briefly, 10 µl cell extract was mixed in triplicate with 90 µl of 50 mM NaCl, 10 mM HEPES, pH 7.0, 40 mM β-glycerophosphate, 2 mM MgCl_2_, 5 mM EGTA, 0.1 mg/ml BSA, 0.1% CHAPS and 10 µM Ac-DEVD-AMC (caspase 3/7 substrate) or 50 µM Ac-LEHD-AMC (caspase 9 substrate; both from Bachem, Weil am Rhein, Germany). Kinetics of substrate cleavage was recorded at 37°C using a Victor V fluorimeter (Perkin Elmer, Rodgau, Germany); the increase in substrate cleavage over time was used to calculate the caspase activity.

### Co-immunoprecipitation

Binding partners of constituents of the apoptosome were identified by co-immunoprecipitation. To this end, 400 µl of cytosolic caspase 3 activation extract were incubated with or without 1 × 10^8^ parasites per ml for 1 hour at room temperature. The caspase cascade was then activated for 15 min at 37°C by addition of 10 µg/ml cytochrome *c,* 250 µM dATP and 250 µM DTT or was left non-activated. An aliquot of 100 µl was then used to measure the caspase activity as described above, and the remaining 300 µl were incubated overnight at 4°C with 1.4 µg of mouse monoclonal anti-Apaf-1 (clone 24; BD Transduction Laboratories, Heidelberg, Germany) or rabbit polyclonal anti-caspase 9 (H-170; Santa Cruz Biotechnology, Heidelberg, Germany) and 100 µM of the caspase 3 inhibitor Ac-DMQD-CHO (Calbiochem, Darmstadt, Germany) to inhibit secondary Apaf-1 cleavage 55. Immune complexes were collected by incubation with 40 µl of 50% Protein A-Sepharose (GE Healthcare, Freiburg, Germany) for 90 min in an end-over-end rotator and subsequent centrifugation. After having been extensively washed, immunoprecipitates were analyzed by immunoblotting.

### SDS-PAGE and Western blotting

Cell extracts, subcellular fractions, immunoprecipitates or recombinant proteins incubated with *T. gondii* lysate were separated by standard SDS-PAGE under reducing conditions. After semi-dry transfer of proteins to NC membranes (Hybond ECL; GE Healthcare, Freiburg, Germany), unspecific binding sites were blocked using 5% dry skimmed milk, 0.2% Tween-20, 0.02% NaN_3_ in PBS, pH 7.4. Membranes were incubated overnight at 4°C with rabbit anti-caspase 3 antiserum (1:200), rabbit anti-caspase 9 antiserum (1:200), 2 µg/ml mouse monoclonal anti-cytochrome* c* (clone 7H8.2C12), 1 µg/ml mouse monoclonal anti-Apaf-1 (clone 24) (all from BD Transduction Laboratories, Heidelberg, Germany), 1 µg/ml mouse monoclonal anti-bovine cytochrome *c*-oxidase subunit IV (clone 20E8-C12; Molecular Probes, Leiden, The Netherlands), rabbit anti-*T. gondii* antiserum, rabbit anti-*T. gondii* surface antigen 1 (TgSAG1) or mouse monoclonal anti-actin (clone C4, kindly provided by J. Lessard, Cincinnati, OH; 1: 10,000) diluted in 5% dry skimmed milk, 0.05% Tween-20 in PBS, pH 7.4. After washing (0.05% Tween-20 in PBS, pH 7.4), primary antibodies were labeled with horseradish peroxidase-conjugated anti-rabbit or anti-mouse IgG (Dianova, Hamburg, Germany). After extensive washing, immune complexes were exposed to enhanced chemiluminescence reagent (GE Healthcare, Freiburg, Germany), and digital images were recorded using a LAS-4000 luminescent image analyzer (Fujifilm, Düsseldorf, Germany).

### Apoptosome formation in a reconstituted *in vitro* system

The impact of *T. gondii* on the apoptosome was also assessed in a reconstituted caspase 9 activation assay 21. To this end, recombinant human Apaf-1 and caspase 9 were expressed in Sf21 insect cells or *E. coli*, respectively, and purified as described 21. A reaction mixture of 0.4 µM Apaf-1 and 0.2 µM caspase 9 in 50 mM HEPES, pH 7.5, 100 mM NaCl, 20 mM MgCl_2_ and 5 mM DTT was incubated or not with 2 - 250 µg/ml *T. gondii* lysate for 1 hour at room temperature. Apoptosome formation was then triggered for 15 min at 30°C using 2 µM cytochrome *c* and 1 mM ATP. After addition of 100 µM Ac-LEHD-AFC (Biomol GmbH, Hamburg, Germany), caspase 9 activity was fluorimetrically measured in a Cary Eclipse fluorescence spectrophotometer (Agilent Technologies, Böblingen, Germany). In order to determine the impact of *T. gondii* on preassembled apoptosomes, 0.4 µM Apaf-1 and 0.2 µM caspase 9, 2 µM cytochrome *c *and 1 mM ATP in 50 mM HEPES, pH 7.5, 100 mM NaCl, 20 mM MgCl_2_ and 5 mM DTT were incubated for 1 hour at room temperature. Thereafter, *T. gondii* lysate was added at the concentrations indicated above and incubated for 45 min at room temperature and subsequently for 15 min at 30°C before measuring the caspase 9 activity as above.

### Statistical analyses

Results are expressed as means ± S.E.M. of three independent experiments unless otherwise stated. Mean values of normally distributed continuous variables were compared using the Student’s *t*-test. P-values of less than 0.05 were considered to be significant.

## SUPPLEMENTAL MATERIAL

Click here for supplemental data file.

All supplemental data for this article are also available online at http://microbialcell.com/researcharticles/toxoplasma-gondii-inhibits-cytochrome-c-induced-caspase-activation-in-its-host-cell-by-interference-with-holo-apoptosome-assembly/.
