# Supplementary material for: Toxoplasma gondii inhibits cytochrome c-induced caspase activation in its host cell by interference with holo-apoptosome assembly
Source: Microb Cell. 2015 May 4;2(5):150–62. doi: 10.15698/mic2015.05.201 (PMC5349237; doi:10.15698/mic2015.05.201)
Supplement: Supplementary file 1 [file mic-02-150-s01.pdf]

Supplemental data: Figure S1

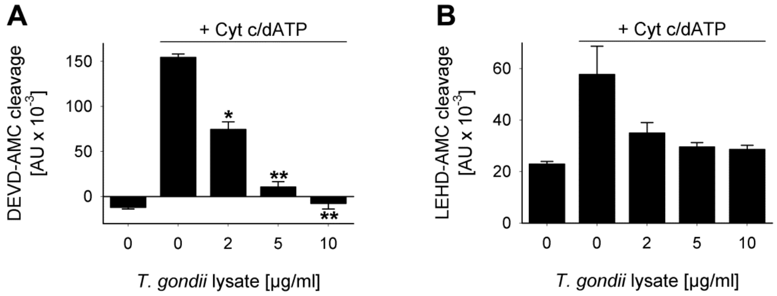

FIGURE S1: Protein extract of *T. gondii* dose-dependently diminishes caspase 3/7 and caspase 9 activities that were triggered in cytosolic T cell extracts by cytochrome c and dATP. (A, B) Cell-free cytosolic extracts of Jurkat cells were incubated with increasing amounts of *T. gondii* lysate or were left untreated. After 1 hour, caspase activation was triggered or not by cytochrome c and dATP as indicated. Cleavage of the caspase 3/7 substrate DEVD-AMC **(A)** or of the caspase 9 substrate LEHD-AMC **(B)** was measured fluorimetrically. Data represent the increase in substrate cleavage over time; they represent means ± S.E.M. from 3 independent experiments. Significant differences were identified by Student's *t*-test (*: $p < 0.05$; **: $p < 0.01$).
